# An aboveground pathogen inhibits belowground rhizobia and arbuscular mycorrhizal fungi in *Phaseolus vulgaris*

**DOI:** 10.1186/s12870-014-0321-4

**Published:** 2014-11-28

**Authors:** Daniel J Ballhorn, Brett S Younginger, Stefanie Kautz

**Affiliations:** Department of Biology, Portland State University, Portland, OR 97201 USA

**Keywords:** *Colletotrichum gloeosporioides*, Induced response, Plant defense, Plant–pathogen interaction, Polyphenol oxidase, Tradeoff

## Abstract

**Background:**

Induced aboveground plant defenses against pathogens can have negative effects on belowground microbial symbionts. While a considerable number of studies have utilized chemical elicitors to experimentally induce such defenses, there is surprisingly little evidence that actual aboveground pathogens affect root-associated microbes. We report here that an aboveground fungal pathogen of common bean (*Phaseolus vulgaris*) induces a defense response that inhibits both the belowground formation of root nodules elicited by rhizobia and the colonization with arbuscular mycorrhizal fungi (AMF).

**Results:**

Foliage of plants inoculated with either rhizobia or AMF was treated with both live *Colletotrichum gloeosporioides*—a generalist hemibiotrophic plant pathogen—and *C. gloeosporioides* fragments. Polyphenol oxidase (PPO), chitinase and β-1,3-glucanase activity in leaves and roots, as well as the number of rhizobia nodules and the extent of AMF colonization, were measured after pathogen treatments. Both the live pathogen and pathogen fragments significantly increased PPO, chitinase and β-1,3-glucanase activity in the leaves, but only PPO activity was increased in roots. The number of rhizobia nodules and the extent of AMF colonization was significantly reduced in treatment plants when compared to controls.

**Conclusion:**

We demonstrate that aboveground fungal pathogens can affect belowground mutualism with two very different types of microbial symbionts—rhizobia and AMF. Our results suggest that systemically induced PPO activity is functionally involved in this above-belowground interaction. We predict that the top-down effects we show here can drastically impact plant performance in soils with limited nutrients and water; abiotic stress conditions usually mitigated by microbial belowground mutualists.

**Electronic supplementary material:**

The online version of this article (doi:10.1186/s12870-014-0321-4) contains supplementary material, which is available to authorized users.

## Background

In their natural environment, plants interact with multiple aboveground and belowground organisms simultaneously [1]. While many of these interactions are beneficial for plants, at the same time plants have to face attack by a broad range of antagonists including pathogenic microbes and herbivores. Simultaneous attack from multiple pathogens or herbivores frequently causes plants to rely on several defensive mechanisms [2]. Many of these plant defenses are inducible, i.e. defensive traits are expressed only after an initial attack by plant antagonists, which is known as induced resistance (IR) [1]. Two commonly studied IR pathways in plants are the jasmonic acid/ethylene-dependent induced systemic resistance (ISR), and salicylic acid-dependent systemic acquired resistance (SAR) [3]. The ISR pathway is induced following damage from necrotrophic pathogens [4], while the SAR pathway is utilized against biotrophic pathogens [5]. Although the utilization of these pathways in response to different antagonists generally holds true, exceptions in nature have been found where nectrotrophic pathogens cause an SAR response in plant hosts [4,5]. The advantage of the ISR and SAR pathways, however, is their facultative mode of expression in response to biotic stress, thus minimizing production costs in plants when defense traits are not needed [6].

Nevertheless, IR may be costly [7]. In addition to the induction of active resistance mechanisms, elicitation of SAR can cause a reduction in plant productivity and plant fitness, which results from a shift in the allocation pathways from plant growth and reproduction to defense [8]. A common further phenomenon associated with this shift from growth to defense is a reduction in photosynthesis [9-12]. Overall, there is substantial evidence that negative effects of foliar resistance expression can result from reduced production or re-allocation of assimilates [13].

Besides creating costs due to resource allocation constraints, defensive traits may negatively affect mutualistic plant symbionts and thus result in ecological costs [14]. When considering plant-associated microbes, some fungi and bacteria include devastating plant antagonists while other species represent key plant mutualists in almost all terrestrial ecosystems [15,16]. Thus, plants have to face the conflict of expressing efficient resistance to pathogens aboveground while maintaining the symbiosis with mutualistic microbes belowground. Given the broad and relatively unspecific character of many anti-pathogen defenses, negative feedback effects on symbiotic microbes seem likely. In fact, several studies have reported such negative effects of IR on microbial plant mutualists through the utilization of acibenzolar-S-methyl (ASM) which chemically induces salicylic acid production [3,17]. Initially marketed to help control powdery mildew in wheat and barley, ASM increases the expression of pathogenesis-related proteins (PR)—including chitinases, peroxidases and β-1-3-glucanase [3]. These proteins can provide resistance to a broad spectrum of future biotic interactions with viruses, bacteria and fungi [18]. However, an inhibition of root nodule formation after chemical induction of pathogen resistance has been shown in several legume species. For example, the application of ASM as either a seed soak or foliar spray to soybean has been shown to cause a decrease in the number of nodules of *Bradyrhizobium japonicum* [3]. Also, the direct application of salicylic acid (SA)—rather than ASM—has demonstrated reduced colonization rates of rhizobia belowground, decreased amounts of leghemoglobin and lower nitrogenase activity overall [19-21]. In terms of interactions with AMF, the picture is less clear [3,22-24], however, overall similar effects seem to be likely [17]. The chemical elicitation of IR through the application of ASM to leaves as a foliar spray or to the roots as a soil drench has been shown to decrease the frequency and extent of AMF colonization [3,17,22-24]. Thus, in terms of ubiquitous plant associations with mutualists, such as rhizobia or AMF, the ISR and SAR pathways may incur fitness costs through the production of defensive compounds intended for plant antagonists that have a deleterious effect on belowground mutualists [3,19-21].

Surprisingly, no study has shown the effects of live natural aboveground fungal pathogens on belowground rhizobial and AMF plant mutualists. Moving away from artificial elicitors to biotic experimental systems is critical to understanding the relevance of such observations in nature. Both rhizobia and AMF are keystone species in almost all terrestrial ecosystems and can alter the plant defensive phenotype with cascading effects for herbivores [25-27]. Given the ubiquitous occurrence of fungal plant pathogens, there is a remarkable lack of knowledge. In order to contribute to filling this gap, we utilized a natural plant-pathogen-rhizobia-AMF system to uncover the effects of aboveground pathogens on defensive plant traits and two types of belowground mutualists. Specifically, in our experiments we treated common bean plants (*Phaseolus vulgaris*) with a foliar pathogen (*Colletotrichum gloeosporioides*). The cosmopolitan fungus, *Colletotrichum gloeosporioides*, is a facultative generalist hemibiotrophic plant pathogen. Infection of plant tissue occurs through wounds or stomata as well as via penetration of intact plant surfaces [28,29]. At this stage, the fungus lives biotrophically and develops primary infection hyphae [30,31]. When the host tissue is destroyed in the course of an infection, the fungus develops secondary necrotrophic hyphae and produces new spores after a few days. Since *Colletotrichum gloeosporioides* exists initially as a biotroph and later as a necrotroph, different plant defense mechanisms may be induced over the course of an infection. An initial biotrophic infection by the fungus should result in an upregulation of salicylic acid, characteristic of systemic acquired resistance (SAR) and associated PR proteins, e.g. chitinases and β-1,3-glucanase. However, after development of necrotrophic hyphae within plant tissue, the jasmonic acid/ethylene-dependent induced systemic resistance (ISR) pathway should increase in expression along with its associated PR proteins, e.g. polyphenol oxidase (PPO) [4,5,32,33]. In response to *C. gloeosporioides* treatments, we measured PPO, chitinase and β-1,3-glucanase activity as key PR proteins involved in plant response to pathogen attack [18,34], and quantified the simultaneous belowground colonization with rhizobia and AMF in pathogen-inoculated plants. To disentangle effects of reduced photosynthetic leaf area due to the formation of fungal lesions from effects of enhanced PPO activity, we also applied fungal cell fragments—not resulting in lesions but inducing PPO activity—in defined concentrations and measured the effects on AMF and rhizobial colonization. To the best of our knowledge, our study is the first to functionally address the effects of aboveground pathogens on belowground microbial symbionts.

## Results

### Preliminary rhizobia and AMF colonization experiment

**Figure 1:**
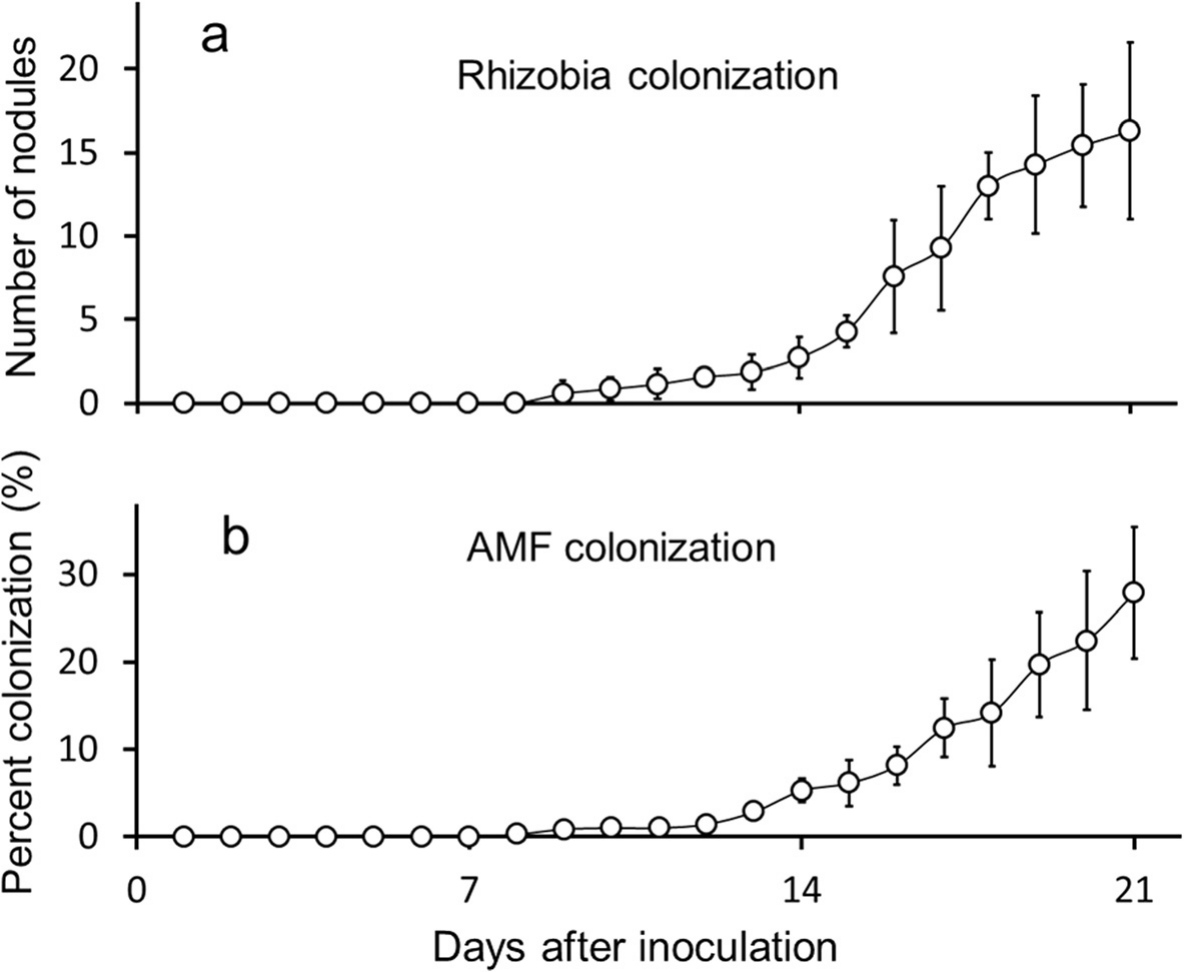
**Preliminary quantification of root-associated microbes.** After destructive harvest of plants (n =7 plants per day; n =147 plants total) root colonization with **(a)** rhizobia (number of nodules) and **(b)** percent of roots showing arbuscular mycorrhizal fungi (AMF) colonization were quantified over a time period of 21 days. Values shown are means ± SD.

**Figure 2:**
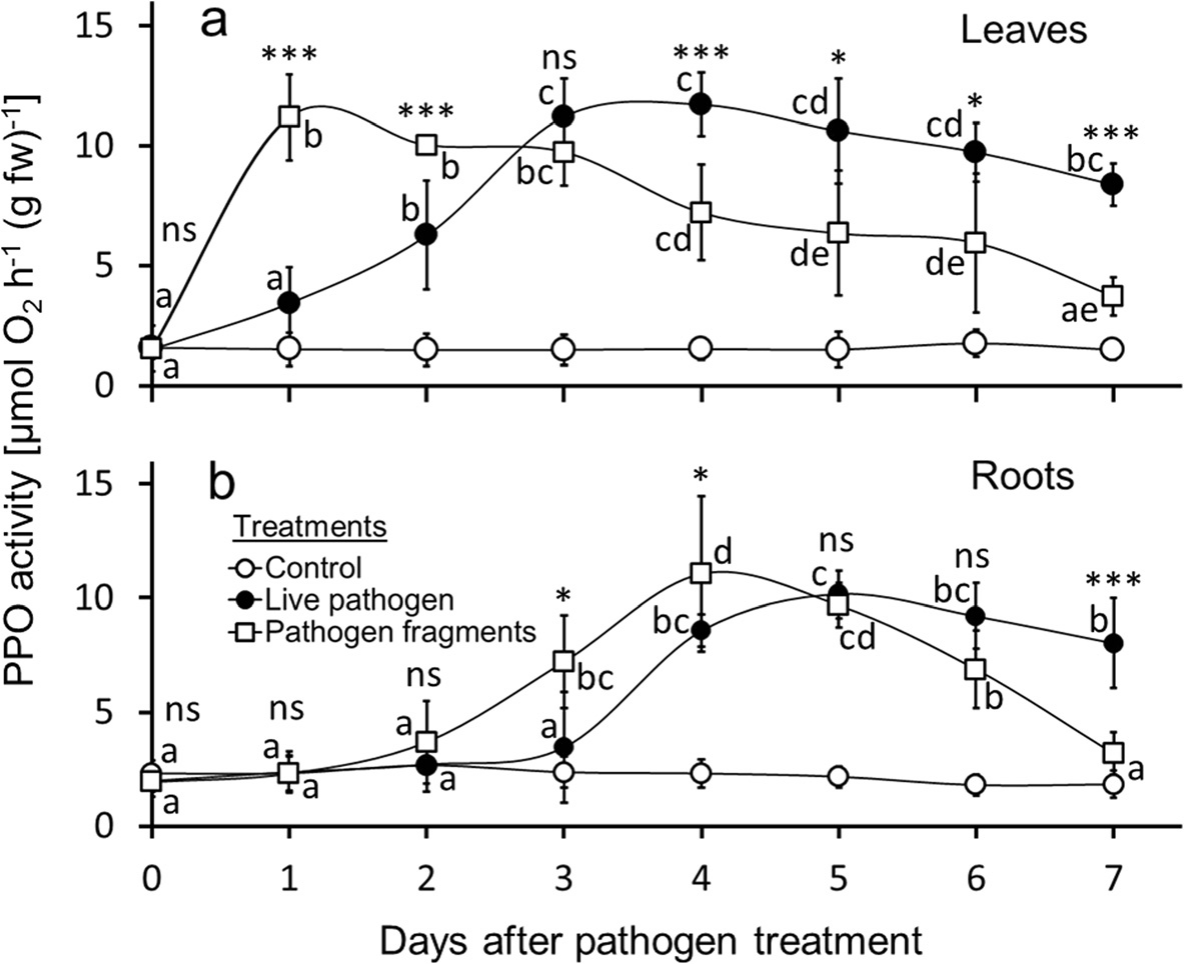
**PPO activity in leaves and roots of common bean following treatment with live pathogen and pathogen fragments.** Foliage of common bean (*Phaseolus vulgaris*) was inoculated with live *Colletotrichum gloeosporioides* and treated with *C. gloeosporioides* fragments. Polyphenol oxidase (PPO) activity in leaves **(a)** and roots **(b)** was measured (n =9 plants per day; 63 plants total per treatment) over an experimental period of 7 days. Values shown are means ± SD. Letters next to data points indicate significant differences within each pathogen treatment group according to post-hoc analysis (Tukey’s HSD; *P* <0.05) after one-way ANOVA. ***P <0.001, *P <0.05, ns *P* >0.05 between treatment groups according to t-test.

**Figure 3:**
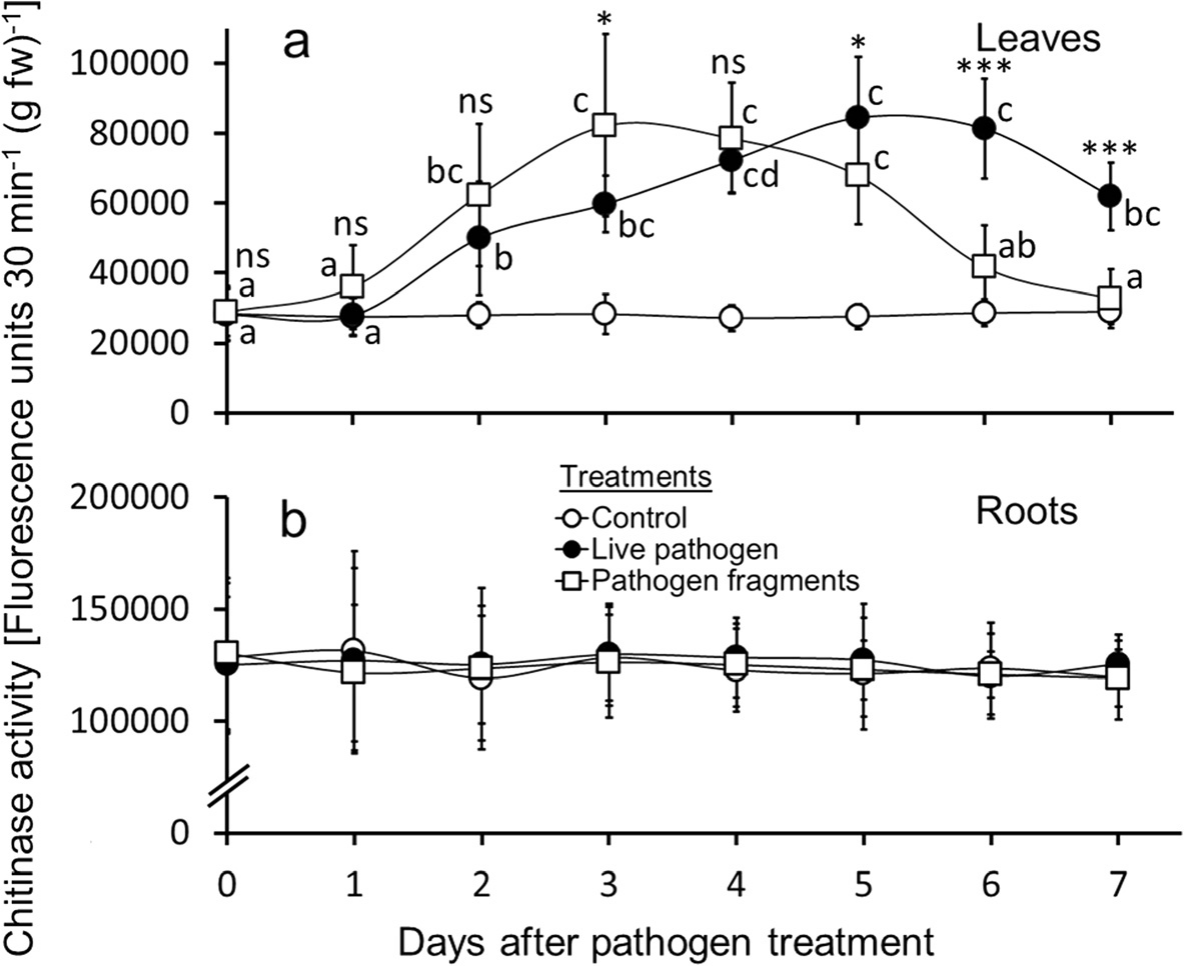
**Chitinase activity in leaves and roots of common bean following treatment with live pathogen and pathogen fragments.** Foliage of common bean (*Phaseolus vulgaris*) was inoculated with live *Colletotrichum gloeosporioides* and treated with *C. gloeosporioides* fragments. Chitinase activity in leaves **(a)** and roots **(b)** was measured (n =9 plants per day; 63 plants total per treatment) over an experimental period of 7 days. Values shown are means ± SD. Letters next to data points indicate significant differences within each pathogen treatment group according to post-hoc analysis (Tukey’s HSD; *P* <0.05) after one-way ANOVA. ***P <0.001, *P <0.05, ns *P* >0.05 between treatment groups according to t-test.

**Figure 4:**
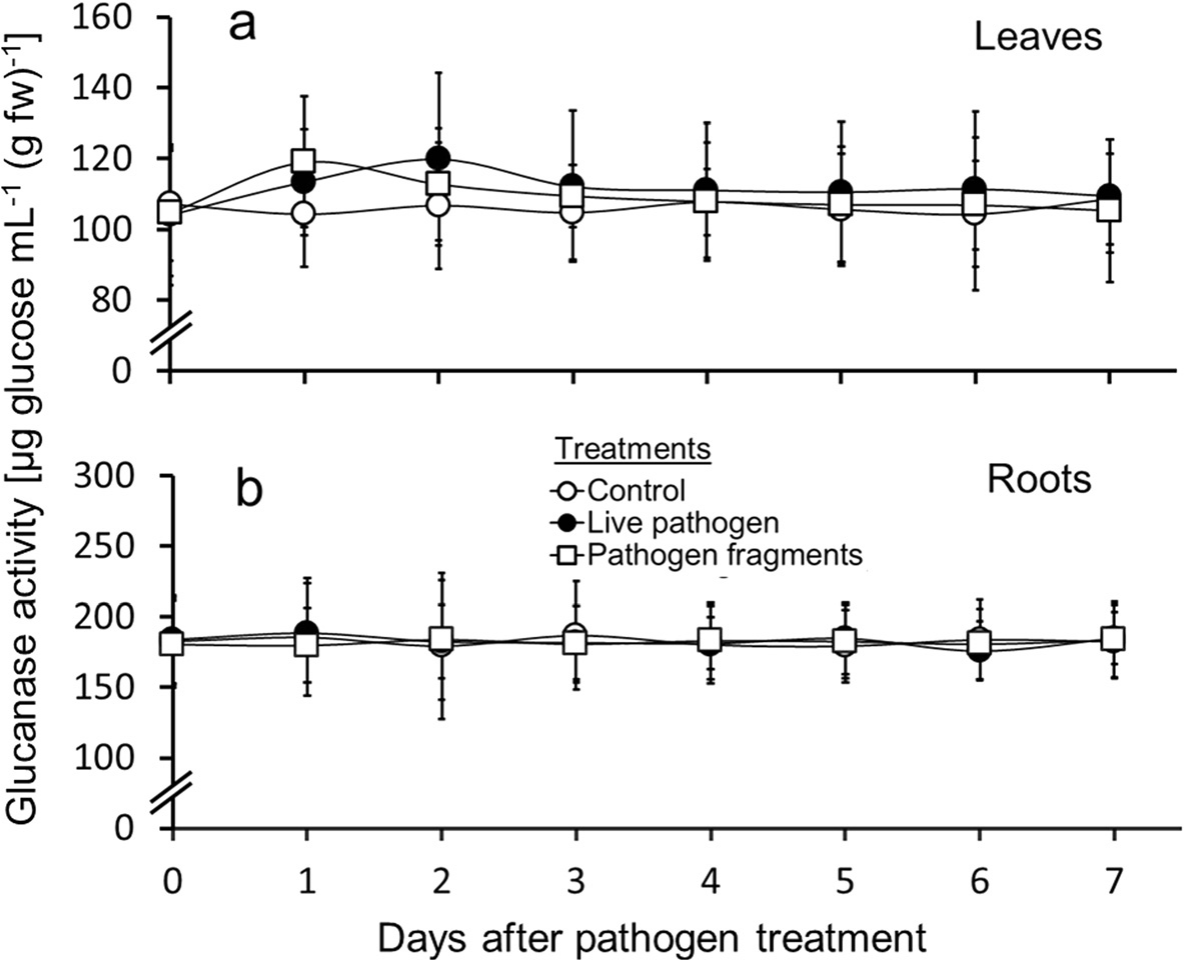
**β-1,3-glucanase activity in leaves and roots of common bean following treatment with live pathogen and pathogen fragments.** Foliage of common bean (*Phaseolus vulgaris*) was inoculated with live *Colletotrichum gloeosporioides* and treated with *C. gloeosporioides* fragments. β-1,3-glucanase activity in leaves **(a)** and roots **(b)** was measured (n =9 plants per day; 63 plants total per treatment) over an experimental period of 7 days. Values shown are means ± SD. There were no significant differences within each pathogen treatment group according to post-hoc analysis (Tukey’s HSD; *P* <0.05) after one-way ANOVA and no significant differences between treatments according to t-test.

**Figure 5:**
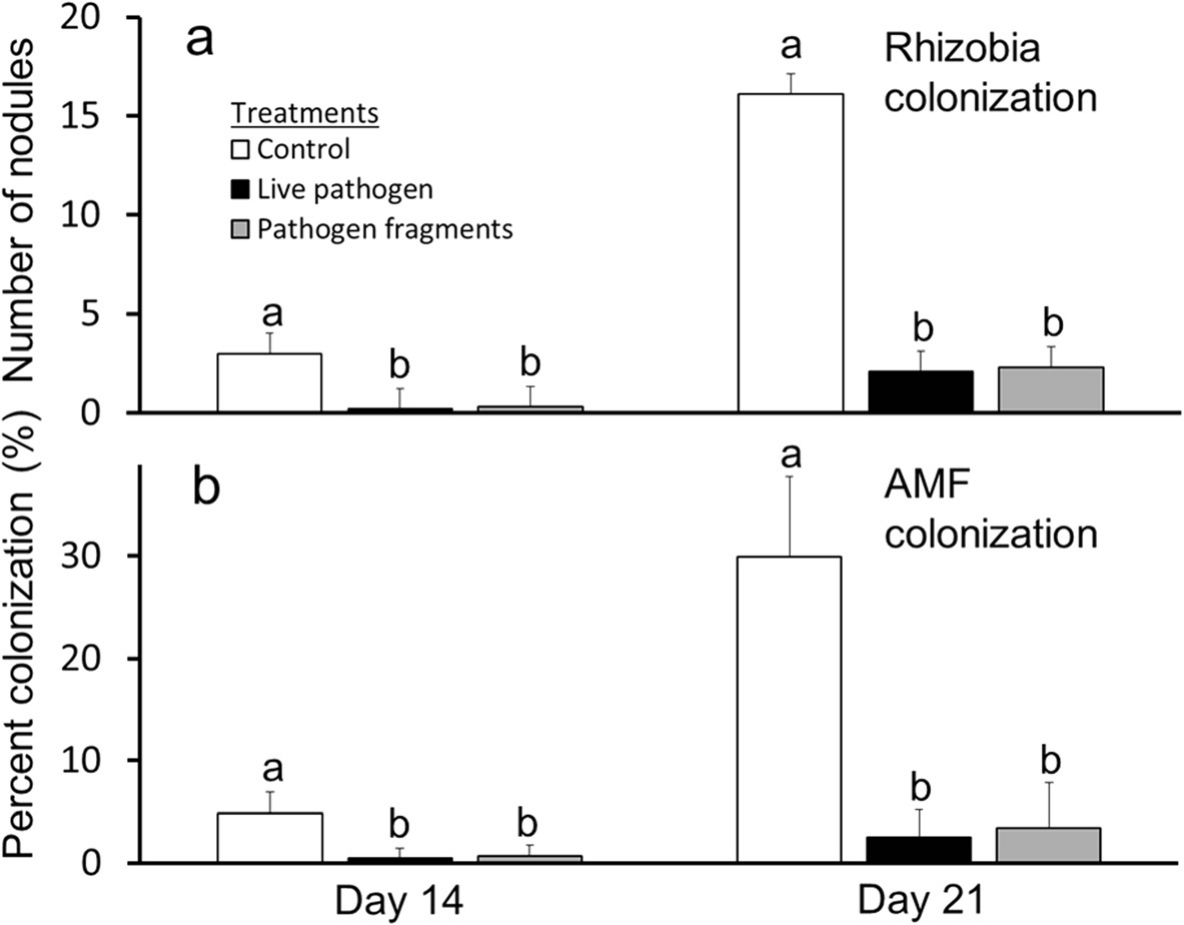
**Effect of live pathogen and pathogen fragments on root colonization with rhizobia and arbuscular mycorrhizal fungi.** Common bean plants were inoculated with rhizobia and AMF. Seven days after rhizobial and AMF inoculated, plants were treated with live *Colletotrichum gloeosporioides* and *C. gloeosporioides* fragments. At day 14 and 21 after pathogen treatments colonization of plant roots with **(a)** rhizobia (number of root nodules) and **(b)** percent of roots showing arbuscular mycorrhiza (AMF) colonization were quantified. Values shown are means ± SD; n =9 plants per day and treatment. Letters on top of the columns indicate significant differences among pathogen treatment groups according to post-hoc analysis (Tukey’s HSD; P <0.05) after one-way ANOVA.

Plants used for the initial evaluation of colonization rates with rhizobia and AMF (Figure 1, Additional file 1) and experimental plants (including controls) used for inoculations and pathogen treatment experiments (Figures 2, 3, 4 and 5, Additional files 2, 3, 4 and 5) belonged to different sets of plants. Plants used for preliminary colonization observations were cultivated about a month before the set of plants used in the pathogen treatments because of logistical reasons. In the preliminary colonization trials, rhizobial nodules started to develop 9 days after inoculation with an average number of 16.3 nodules per plant by day 21 (n =7 plants destructively harvested per day; 147 total) (Figure 1a). Microscopic quantification of mycorrhizal fungi (AMF) colonization showed an initial appearance of AMF structures at day 9, as well. The average percentage of roots colonized by AMF at day 21 was 27.9% (n =7 plants analyzed per day; 147 total) (Figure 1b).

### PPO activity—leaves

We observed significant changes over time in the activity of polyphenol oxidase (PPO) [μmol O_2_ h^−1^ (g fw)^−1^] in leaves of plants induced with both live *C. gloeosporioides* (*F*_7,64_ = 54.533; *P* <0.001) and *C. gloeosporioides* fragments (*F*_7,64_ = 30.253; *P* <0.001) (n =9 plants analyzed per day; 63 total) (Figure 2a, Additional file 3). PPO activity in control plants treated with water instead of spore suspension or fungal fragments showed low and very similar PPO activity throughout the experiment (*F*_7,64_ = 0.232; *P* =0.976). In leaves inoculated with the live fungus, the increase in PPO activity was significant after two days and showed a further significant increase at day three. Enzymatic activity started to decline at day five and decreased further over the course of the experiment. Leaves inoculated with pathogen fragments showed a faster increase in PPO-activity than leaves inoculated with the live fungus. PPO activity was significantly enhanced at day one and decreased after this peak over the time of the experiment. Compared to the peak of activity, we observed a significant reduction at day four with a further significant decrease at day seven. Thus, the increase in PPO activity in leaves in response to live pathogen inoculation was delayed compared to treatments with fungal fragments, but PPO activity remained at elevated levels—above levels observed for fungal fragments—for an extended period of time until the end of the experiment. When comparing both treatments (live pathogen vs. fungal fragments) at day one and two, leaves induced with fungal fragments showed significantly higher PPO activity than leaves inoculated with the live pathogen (Figure 2a). While at day three no significant differences in PPO activity were measured between the treatments, we observed significantly higher enzyme activities in leaves inoculated with the live pathogen at days four, five, six and seven.

### PPO activity—roots

The activity of PPOs in roots showed significant variation in response to treatments with both the live pathogen (*F*_7,64_ = 54.323; *P* <0.001) and fungal fragments (*F*_7,64_ = 35.567; *P* <0.001). The pattern of PPO expression in roots was similar to leaves with a slower but more sustainable increase in plants inoculated with live pathogen suspension compared to a stronger increase and faster decline with near baseline levels by day seven in plants treated with fungal fragments (n = 9 plants analyzed per day; 63 total). Compared to the controls, the increase in PPO activity became significant at days four (live pathogen) and three (fungal fragments) and increased further at days five and four, respectively. For plants induced with fungal fragments, we observed a significant decrease at day six and further on day seven compared to the peak of activity at day four. Leaves inoculated with fungal fragments showed a moderate, non-significant decrease of PPO activity until the end of the experiment at day seven. In general, the induction of PPO activity in roots showed a characteristic time lag compared to leaves as the maximum activities were reached five days after inoculation with live pathogen and four days after application of pathogen fragments (Figure 2b, Additional file 4). However, the maximum PPO activity in roots was only slightly lower than those in leaves.

### Chitinase activity—leaves

Inoculation of leaves with live *C. gloeosporioides* (*F*_7,64_ = 31.162; *P* <0.001) and fungal fragments (*F*_7,64_ = 17.035; *P* <0.001) both had significant effects on chitinase activity in leaves of *P. vulgaris* (Figure 3a, Additional file 3). Control plants showed no increase in leaf chitinase levels throughout the experiment. In the live pathogen treatment, PPO activity first became significantly increased at day two compared to the controls. We observed further increases at days three and four with maximum levels reached at day five. PPO activity in these inoculated leaves remained over twice as high as in the control plants after seven days at the end of the experiment. In contrast to the live pathogen treatment, PPO activity in leaves induced with fungal fragments increased rapidly, reached its peak at day three and declined to baseline levels towards the end of the experimental period. In fragment-induced leaves, the increase in PPO activity was significant at day two and showed a significant decline at day six.

### Chitinase activity—roots

In contrast to *P. vulgaris* leaves, root chitinase activity showed no significant change in response to either foliar inoculation with live the pathogen or to leaf treatments with pathogen fragments (Figure 3b, Additional file 4).

### β-1,3-glucanase activity—leaves

Treatment of leaves with both the live and fragmented fungus increased β-1,3-glucanase activity in leaves with peaks at day two for live pathogens and day one in the case of pathogen fragments. However, changes in β-1,3-glucanase activity were not significant (Figure 4a, Additional file 3). Although not significant, the overall pattern of changes in β-1,3-glucanase activity corresponded to the other pathogen induced enzyme activities (PPO and chitinase) with fragments resulting in a faster but more short-lived increase compared to inoculation experiments with live fungi.

### β-1,3-glucanase activity—roots

In a similar manner to leaf β-1,3-glucanase activity, no significant difference was observed between pathogen treatments and control plants in the roots (n =9 plants analyzed per day; 63 total) (Figure 4b, Additional file 4). However, root β-1,3-glucanase activity was 50% higher than leaf β-1,3-glucanase activity in pathogen treatment and control groups throughout the experiment.

### Nodulation in plants inoculated with live pathogen and treated with fungal fragments

Both the live pathogen and pathogen fragments caused a significant reduction in the number of rhizobia nodules at days 14 and 21 after treatment (n = 9 plants evaluated per day; 18 total) compared to control plants (Figure 5a, Additional file 5). Treatment with live pathogen and pathogen fragments resulted in a significant reduction of 86% and 80%, respectively, in the number of nodules when compared to control plants at day 14 (*F*_2,24_ = 34.343, *P* <0.001) while there was a 77% and 75% reduction in the number of nodules by day 21 for the live pathogen and pathogen fragment treatments, respectively, when compared to control plants (*F*_2,24_ = 47.286, *P* <0.001). To test for the transferability of colonization data from the preliminary experiment (Figure 1a) to the pathogen treatment plants (Figure 5a), we compared rhizobial colonization between both sets of control plants at days 14 and 21. We observed no significant difference in rhizobial colonization between both sets of plants (day 14: t-test, *P* =0.638; day 21: t-test, *P* =0.948).

### AMF colonization in plants inoculated with live pathogen and treated with fungal fragments

The live pathogen and pathogen fragments caused a significant reduction in the percentage of roots colonized by AMF at days 14 and 21 compared to control plants (n = 9 plants evaluated per day; 18 total) (Figure 5b, Additional file 5). Treatment with live pathogen and pathogen fragments resulted in a 79% and 76% reduction, respectively, in the percentage of roots colonized by AMF compared to controls at day 14 (*F*_2,24_ = 25.506, *P* <0.001). There was an 84% and 79% reduction in the percentage of roots colonized by AMF by day 21 when compared to control plants for the live pathogen and pathogen fragment treatments, respectively (*F*_2,24_ = 73.656, *P* <0.001). To test for the transferability of colonization data from the preliminary experiment (Figure 1b) to the pathogen treatment plants (Figure 5b), we compared AMF colonization between both sets of control plants at days 14 and 21. AMF colonization rates were not significantly different in the initial screening for AMF establishment when compared to plants used in the pathogen treatments (day14: t-test, *P* =0.744; day 21: t-test, *P* =0.693).

### Additional experiment examining belowground rhizobia and AMF colonization in plants inoculated with live pathogen and treated with fungal fragments

In an additional, timely independent experiment that re-examined belowground colonization rates, both the live pathogen and pathogen fragments caused a significant reduction nodules at days 14 and 21 after treatment (n = 9 plants evaluated per day; 18 total) compared to control plants [see Additional files 2 and 5]. The live pathogen and pathogen fragments also caused a significant reduction in the percentage of roots colonized by AMF at days 14 and 21 compared to control plants (n = 9 plants evaluated per day; 18 total). These results were very similar to patterns seen in the initial examination of belowground colonization rates of rhizobia and AMF.

### Correlations in enzyme activity and belowground colonization

A negative correlation existed between PPO activity in the roots and rates of belowground colonization with rhizobia (r = -0.893, *P* <0.001) and AMF (r = -0.895, *P* <0.001) (Pearson’s two-tailed correlation) (Table 1). However, no significant correlations existed between chitinase activity and rhizobia colonization (r = 0.016, *P* = 0.939) or chitinase activity and AMF colonization (r = -0.013, *P* =0.950) belowground. Furthermore, β-1,3-glucanase activity in the roots showed no significant correlations to rhizobia colonization (r = -0.163, *P* = 0.417) or AMF colonization (r = 0.061, *P* = 0.764) (Table 1).

**Table 1:** **Correlation between root enzyme activity and belowground colonization with rhizobia and arbuscular mycorrhizal fungi (AMF)**

	**Rhizobia**		**AMF**	
	***r***	***P***	***r***	***P***
**PPO**	**−0.893**	**< 0.001**	**−0.895**	**< 0.001**
**Chitinase**	0.016	0.939	−0.013	0.95
**β-1,3-glucanase**	−0.163	0.417	0.061	0.764

Pearson two-tailed coefficients (*r*) in bold are statistically significant at the 0.05 level.

## Discussion

Plant defense mechanisms expressed in response to aboveground attack by pathogens or herbivores can affect belowground mutualism with beneficial microbes. This interaction between defense and symbiosis can have significant effects on a plant’s ability to obtain nutrients and water through their beneficial microbial symbionts. Previous studies have shown a decrease in both rhizobia and arbuscular mycorrhizal fungi (AMF) colonization following the application of acibenzolar-S-methyl (ASM) as a foliar spray to soybean [3,17,23]. However, the chemical elicitation of defense-related mechanisms using artificial agents may not truly mimic a plant’s ability to protect itself from pathogenic fungi in nature, nor show a realistic inhibition of belowground microbes. In contrast to induction with ASM, an induction of induced resistance (IR) through live pathogens, with implications for belowground mutualistic microbes, has yet to be demonstrated to the best of our knowledge. Analyzing this type of biotic defense stimulation is relevant to understanding processes in natural systems.

It has previously been shown that plants also respond to aboveground phloem-feeding insects by increasing the expression of pathogenesis-related proteins (PR). These plant responses subsequently affected above- and belowground symbiotic microbe communities, albeit with mixed results. Mayer *et al.* [35] found increased resistance to powdery mildew in the leaves of tomato plants following silverleaf whitefly feeding. Conversely, Yang *et al.* [36] demonstrated a positive effect on belowground bacterial and fungal microbes when plants were experimentally exposed to whitefly feeding. Our study is the first to utilize both live fungal cells and fragments from fungi to induce plant defense mechanisms resulting in reduced belowground mutualism. Furthermore, we showed that both experimental inoculation with live pathogens and treatment with pathogen fragments resulted in a comparable decrease in the interaction with two very different microbes of the rhizosphere simultaneously, rhizobia and AMF; a novel finding which has not been observed previously (but see Faessel *et al.* [3] where application of ASM significantly reduced rhizobia colonization and also reduced the intensity of mycorrhizal fungi colonization and the proportion of arbuscules).

In our study, we observed strong effects of pathogen-induced defenses on the intensity of both types of belowground mutualists. The number of rhizobia nodules on *Phaseolus vulgaris* plant roots was significantly reduced when plants were treated with both the live fungal pathogen and fungal fragments when compared to controls (Figure 5a). Similarly, the percentage of roots colonized by AMF was significantly lower in bean plants treated with live pathogen and fungal fragments (Figure 5b).

Our data suggest that the effects of induced resistance (IR) may have resulted from increased polyphenol oxidase (PPO) activity in pathogen treated plants (Table 1). In our plant system, the increase in PPO activity was the only parameter measured which responded on a systemic level. In response to the pathogen treatments, we observed an increase in foliar PPO activity, which was particularly rapid with pathogen fragments. In the roots we observed a similar response. However, the increase in PPO activity in roots was delayed by 1-2 days; indicative of a systemic transfer of a signal, likely salicylic acid [20,21]. Previous studies on lima bean (*Phaseolus lunatus*) showed a high efficiency of PPO activity as defense against *Colletotrichum gloeosporioides* supporting a critical role of PPOs in the defense response to *C. gloeosporioides* also in this plant system [37,38]. PPOs—largely stored in the thylakoid lumen until liberation from tissue damage or pathogen attack—play a key role in the oxidation of intracellular phenolics to reactive quinones. These quinones are highly oxidizing and generate reactive oxygen species (ROS), serving as an effective defense to pathogens. Furthermore, reactive quinones can reduce the protein availability within plant tissues through alkylation rendering them less nutritive to pathogens and herbivores [39,40]. It is likely that reduced colonization rates belowground with rhizobia and AMF were the result of oxidation from PPO activity. In contrast to PPO activity, we did not detect systemic responses in terms of elevated chitinase or β-1,3-glucanase activity. Both enzyme activities showed a distinct response to both treatments (live pathogen and pathogen fragments) in leaves (Figures 2a and 3a), but activities in roots remained unchanged over the experimental period (Figures 2b and 3b). However, a systemic increase of PPO activity in both the leaves and roots, most likely resulting from salicylic acid activity, should have also resulted in an increase in chitinase and β-1,3-glucanase activity in the roots [34]. It is possible that the initial colonization with microbial mutualists—rhizobia and AMF—caused increases in these defensive proteins in the roots of both pathogen treatment and control plants. Further induction of chitinase and β-1,3-glucanase activity in only the leaves could have been the result of pathogen and pathogen fragment treatments aboveground.

Genotypic differences in iridoid glycosides (IG) utilized during plant allelopathy may have also caused reduced colonization rates belowground in our experiment. Accessions of *Plantago* that produce higher levels of the IG compounds aucubin and catalpol demonstrate reduced levels of AMF colonization [41]. Initial colonization of *Plantago* roots by AMF can also cause increases in aucubin concentrations. However, we did not analyze intrinsic differences in IG production in our *Phaseoulus vulgaris* plants and cannot draw further conclusions on the likelihood of its effect on reduced colonization. The mechanical damage caused during inoculation with live pathogens and pathogen fragments from the glass pin may have also caused variations in belowground AMF colonization. *Medicago* plants subjected to repeated wounding have demonstrated increases in AMF abundance and lower susceptibility to fungal pathogens through an upregulation of jasmonic acid [42]. The possibility exists that certain genotypes of *P. vulgaris* respond to mechanical wounding in varying degrees, thus influencing the extent of belowground AMF colonization. Further analyses of jasmonic acid production in response to wounding is needed to confirm this effect with *P. vulgaris*.

In addition to direct impacts of IR on microbial root symbionts, shifts in the allocation pathways from plant growth to defense may also cause reduced colonization with rhizobia or AMF. Furthermore, a reduction in photosynthesis may limit the plants’ ability to both grow vegetatively and form root symbioses [9-12]. In addition to cellular processes leading to reduced photosynthesis, the mere loss of photosynthetic leaf area due to the formation of pathogen lesions may also reduce the availability of a plant’s photosynthates. For example, in a recent study we could show a quantitative effect of leaf area loss on the production of extrafloral nectar in lima bean (*P. lunatus*), which is mainly composed of sugars directly derived from photosynthesis [43]. Thus, there is evidence that negative effects of induced resistance can result from reduced production or re-allocation of assimilates [13]. However, as we observed similar effects for treatments with live pathogens and fungal fragments on both belowground symbioses, we could rule out the possibility that photosynthetic leaf area caused the observed effects. While treatments with live fungal pathogens resulted in the formation of lesions and clearly reduced photosynthetic leaf area, the treatment with fungal fragments did not cause any visible leaf damage, but resulted in similar effects both in terms of PPO induction and reduced intensity of microbial symbioses. However, the possibility still exists that root colonization from both types of microbes was inhibited by a resource allocation towards defensive compounds. It would be interesting to note reductions in biomass resulting from pathogen treatments in plants not harboring belowground microbes. An assessment of the re-allocation of photosynthate away from growth and towards defense could provide further evidence of whether defensive compounds were the causative agent behind reduced belowground colonization rates [1,14,38]. Both rhizobia and AMF cause a significant carbon sink in plant hosts. As much as 20% of all photosynthate produced in the plant can be consumed by bacterial/fungal associates [44]. A reallocation of photosynthate away from the microbial symbionts and towards the production of defensive compounds may have made the plants less suitable hosts. The likelihood of microbial mutualists affecting induced resistance traits in plants also exists. Future investigations should analyze levels of the same enzyme activity (i.e. PPO, β-1,3-glucanase and chitinase) in plants not inoculated with rhizobia or AMF after treatment with aboveground pathogens. These additional pathogen treatments would help to compare induced resistance traits in plants not harboring belowground mutualists, although these conditions are unlikely to exist in nature [45]. Since β-1,3-glucanase and chitinase activity in the roots did not change following treatment with live pathogens and pathogen fragments, but existed throughout experimentation in higher levels than in leaves, it is possible that inoculations with rhizobia and AMF caused an induction of these defensive compounds in the roots. Conversely, PPO activities in the leaves and roots were more similar following aboveground pathogen treatment, characteristic of a systemic induction.

Previous work has demonstrated a lower susceptibility to fungal pathogens in mutant plants that constitutively express PPO genes [39,46]. Our results suggest that induction of PPO activity in plants from pathogenic attack aboveground extends to the belowground community. This can have serious ramifications on plants under pathogen attack that rely on mutualists in the rhizosphere for nutrient and water acquisition. Although our work is the first to demonstrate an increased expression of PPO following treatment with pathogenic fungi with a concomitant decrease in belowground mutualism, several directions need further investigation.

Although our results on decreased AMF colonization were similar to those of de Román *et al.* [17] and Tosi & Zazzerini [23], their results showed a transient effect on AMF colonization over a period of 14 days after the chemical elicitation of IR with both a foliar spray and seed soak. Our results over 21 days showed a minimal increase in rates of root colonization after initial suppression. Therefore, future studies should look at a longer period of time following fungal elicitation of PPO activity. Furthermore, de Román *et al.* [17] and Tosi & Zazzerini [23] examined chitinase and β-1,3-glucanase levels and found increases in enzyme activity in only the leaves, not the roots. Neither study examined PPO activity in response to chemical elicitation. PPO activity resulting from fungal fragment treatments in our study returned to levels near control plants in both roots and leaves after 7 days. This warrants further investigation into the durability or residual nature of PPOs in roots.

## Conclusion

Here, we show that the expression of polyphenol oxidases (PPOs) as an effective anti-pathogen defense may significantly inhibit the ability of plants to form belowground symbioses. To the best of our knowledge, our results are the first to demonstrate that plant defense responses to aboveground pathogen attack hinder associations with belowground rhizobia and mycorrhizal fungi. In our study, induced PPO activity was observed in plants exposed to fungal pathogens and is most likely the mechanistic basis for this phenomenon. Although plants in nature may already exist in symbiotic associations when confronted with pathogenic fungal attack, the production of defensive compounds (such as PPOs) could decrease or eliminate mutualistic microbes. Plants grown in an agricultural setting may not have time to establish significant associations with mutualists belowground prior to pathogenic attack, particularly in regularly cultivated soils. Our results more accurately mirror circumstances encountered in agriculture; young plants without significant belowground associations under attack from pathogens. Therefore, our results should be considered highly relevant to crops that are dependent upon mutualistic associations and, at the same time, susceptible to fungal pathogens in nutrient and water-limited soils. The complex interplay of defense and mutualism can largely determine the outcome of plant fitness and, given the ubiquitous occurrence of plant pathogens, has broad relevance for plant productivity in natural and agricultural ecosystems.

## Methods

### Plants

Common bean plants (*Phaseolus vulgaris* L. ‘Bush Blue Lake’) were grown from seeds (American Meadows Inc., Williston, VT). Seeds were pre-germinated in sterilized wet paper towels and used for planting when they had developed a 0.5 cm long radicula. Plants were cultivated in a greenhouse with a light regime of 13:11 h in the light:dark period. Light in the greenhouse was provided by a combination (1:1) of HQI-BT 400 W (Osram) and RNP-T⁄LR 400 W (Radium) lamps. Light availability was measured at noon on a sunny day (+ additional lighting) and at table height resulting in an average of 625 μmol photon s^−1^ m^−2^ in full light (LI-250 light meter; LI-COR Biosciences, Lincoln, Nebraska, USA). Temperature was 27:19°C (light:dark) and we maintained a relative air humidity of 70–80%. Plants were grown in containers (one plant per pot) with 12 cm diameter in Sunshine Mix #1, LC1 (peat moss, coarse perlite, starter nutrients with gypsum, dolomitic limestone and wetting agent; SunGro Horticulture®, Bellevue, WA) 175 g per pot and were watered daily.

### Rhizobia

Common bean forms a close association with N_2_-fixing soil bacteria of the family Rhizobiaceae. Commercial inoculum specific to *Phaseolus vulgaris* (*Rhizobium leguminosarum* biovar *phaseoli*) was obtained from INTX microbials LLC, Kentland, IN. In our study, bacteria were cultivated in liquid medium (pH 7.0) containing 1 g yeast extract (Merck, NY), 10 g mannite (Merck, NY), 800 mL deionized water and 200 mL soil extract. The soil extract was prepared from 160 g dry, non-fertilized loamy soil (taken from a fallow grass-covered area) that was suspended in 400 mL deionized water under addition of 0.4 g sodium carbonate and autoclaved at 121°C for 30 min at a pressure of 1,260 mbar. Three days prior to plant inoculation, rhizobia were cultivated at 28°C and 180 rpm on a laboratory shaker (Eppendorf, Westbury, NY). The bacteria solution was then diluted with tap water in a ratio of 1:10 and plants were watered with 100 mL of this solution. Rhizobial inoculum was applied one day after pre-germinated bean seeds were planted.

### Arbuscular mycorrhizal fungi (AMF)

Experimental plants were inoculated with commercial AMF inoculum [powder inoculant, Bio Organics™, La Pine, Oregon (*Glomus aggregatum*, *G. deserticola*, *G. monosporum, Rhizophagus clarus, R. irregularis, Claroideoglomus etunicatum*, *Funneliformis mosseae*, *Gigaspora margarita*, *Paraglomus brasilianum*), 10 cc (8 g) per plant]. AMF inoculum was applied at the same time as rhizobia by making four holes (of approx. 5 cm in depth) in the substrate around the planted seed using a sterile glass Pasteur pipette. In each of the holes we filled 2 g of inoculum powder and plants were slightly watered.

### Fungal pathogen treatments

The strain 62146 of *Colletotrichum gloeosporiodes* (Penzig) Penzig & Saccardo used in this study was obtained from the Leibniz Institute-DSMZ German Collection of Microorganisms and Cell Cultures (Braunschweig, Germany) and proved to be successful in readily colonizing *Phaseolus* sp. leaf tissue in our previous studies [37,38]. *Colletotrichum gloeosporioides* was cultivated in Petri dishes (9.5 cm in diameter) on oat medium, pH 5.0 (4% (v/v) flour, 1.5% (v/v) agar). Cultures were stored in an incubator at a temperature of 25°C, a relative air humidity of 85%, and under a light regime of 10 min light (150 μmol m^−2^ s^−1^) per 12 h to induce development of spores. When plants had developed unfolded primary leaves (seven days after planting), we applied two different pathogen treatments, one with living fungi (spore suspension) and one with fungal fragments. To inoculate plants with the pathogen, leaflets were treated with a spore suspension adjusted to a concentration of 10^5^ spores mL^−1^. Spore samples were taken from 4-day-old fungal cultures, diluted with 5 mL aqua dest. and mixed three times for 1 min. Microscopic determination of spore concentration was carried out by use of an Improved-Double-Neubauer counting chamber. Seven samples of each suspension were counted under the microscope (diagonal squares were counted in a row). Leaflets were injured on the lower surface (one injury per leaflet) using a 0.9 mm glass pin, and 5 μL of spore suspension was pipetted onto the wound [37]. We also applied fungal cell fragments—not resulting in lesions but inducing plant defense responses. To expose plants to fungal fragments, fungal hyphae together with spores were scraped from culture medium and 1 mg fungal tissue (fresh weight) was ultrasonicated in 1 mL sterile deionized water for 5 min directly before the treatment. For application of fungal fragments each primary leaf was injured as described above for the live pathogen and then the lower leaf surface was sprayed with 2 mL cell fragment suspension per leaf using an air brush. We tested for viability of fragments by plating aliquots of the ultrasonicated suspension on agar plates with malt extract (2% (v/v)). None of the replicates showed any fungal growth within 2 weeks and culture conditions described above. We concluded that ultrasonication is sufficient to prepare fungal suspensions containing no viable cells.

### Quantification of root colonization with rhizobia and mycorrhizal fungi

Establishment of rhizobia was evaluated based on nodulation—the formation of visible root nodules. For quantification of nodulation, roots were destructively harvested, carefully washed and nodules were counted per plant.

Mycorrhizal fungi colonization was evaluated by taking 1 g fresh root samples, from each plant, from 4 separate locations of washed roots. Root segments were placed into histocassettes (VWR, West Chester, PA). All root samples were cleared with 10% (v/v) KOH, acidified in 2% (v/v) HCl, stained with 0.05% (v/v) trypan blue solution, and preserved in lactoglycerol [47]. Roots were cut into 1 cm sections and at least 40 cm of roots from each plant were placed on a single microscope slide with lactoglycerol. Microscopic observations were conducted using an AmScope FM320 Trinocular Microscope in both 100x and 400x magnification. Roots were examined for mycorrhizal fungi structures that intersected the microscope eyepiece crosshair at 100 random points using the Magnified Intersections Method [48]. The presence or absence of mycorrhizal fungi structures at 100 intersects was used to calculate percent root length colonization by mycorrhizal fungi per plant [49]. A second experiment was conducted to compare the initial results of belowground colonization rates of rhizobia and AMF in response to treatments with fungal pathogens and pathogen fragments.

### Quantification of polyphenol oxidase activity

All enzymatic assays were conducted using the same individual primary leaf to reduce variation in leaf traits. Per leaf we excised three leaf discs (2 cm diameter) which were immediately weighed (XA204 Delta Range, Mettler Toledo, Switzerland) and transferred to pre-cooled (on ice) 2 ml Eppendorf® tubes. Tubes were placed in a dry shipper filled with liquid nitrogen, transported to the lab and processed within 24 h after sampling. Root samples (three samples of 0.5 g lateral, washed roots per plant from the same part of the root system) were treated identically. We quantified enzymatic activity of polyphenol oxidases (PPOs) by measuring the O_2_ consumption during the oxidation of polyphenols and their derivatives to quinones [50]. For extraction of PPOs, leaflets or roots were homogenized in the threefold volume (v fresh weight^−1^) Sörensen buffer using micro pistils. Extraction was carried out in Eppendorf® tubes (1.5 mL) at 25°C. Subsequently, samples were centrifuged (8000 x g, 10 min, 4°C) and the supernatants were used for analysis of PPO activity. The activity was determined polarographically, using the Clark electrode system (Yellow Springs Instruments, Yellow Springs, OH, USA), by measuring the concomitant oxygen depletion. The electrode was calibrated to 100% O_2_ saturation using O_2_-saturated Sörensen buffer (phosphate–citrate buffer, pH 5.6). We used 4-methylcathechol (Merck, NY) as a standard. The autoxidation of 4-methylcathechol was measured under stirring before each PPO activity analysis. The solution for measuring autoxidation consisted of 2.9 mL Sörensen buffer and 100 μL 4-methylcatechol (225 mM L^−1^). The solution in the O_2_ electrode consisted of 100 μL supernatant and 2.8 mL O_2_-saturated buffer. After 1 min of stirring, 100 μL of 4-methylcatechol (225 mM L^−1^) was added and O_2_ depletion was determined polarographically over a time period of 6 min. This procedure gives relative values of O_2_ depletion including enzymatic O_2_ depletion in the course of substrate oxidation, autoxidation of the substrate, and other O_2_ consuming processes in the plant extracts. Thus, we subtracted both values of autoxidation of the substrate and O_2_ consumption of the samples (measured in the O_2_ electrode without sample or substrate solution, respectively) from total O_2_ depletion.

### Chitinase activity assay

Crude homogenates for chitinase and *β*-1,3-glucanase activity assays were prepared by grinding plant and root samples in liquid nitrogen with a micro pistil and homogenized in 0.1 M sodium citrate buffer, pH 5.0, at a ratio of 1:2 (w/v). The homogenate was centrifuged (10 min, 10,000 x g; Eppendorf 5810R), and the supernatant was used as crude enzyme preparation [51]. Chitinase activity was determined with a fluorimetric assay according to Ren *et al*. [52]. Five μL of methylumbelliferyl β-d-*N, N’,N’*-triacetylchitotrioside hydrate (0.5 mg mL^−1^; Sigma) was added to 95 μL plant extract (leaf root and samples) in a black 96-well microplate. Samples were incubated at 40°C for 30 min in a shaker. The fluorescence was measured using a Molecular Devices SpectraMax Plus^384^ Microplate Reader (excitation 365 nm, emission 450 nm). All values were reported as fluorescence units per gram fresh weight. Extraction buffer served as reference.

### β-1,3-glucanase activity assay

The activity of β-1,3-glucanase was determined in 96-well microplates using an adaptation of the method described by de Román *et al.* [17] and Somogyi [53]. *Laminaria digitata* laminarin (Sigma) was used as substrate. The total volume of 180 μL reaction preparation contained 50 μL plant extract (leaf and root samples), 10 μL laminarin (20 mg mL^−1^ in 50 mM Na-acetate buffer, pH 5.0), 60 μL copper reactive and 60 μL arsenic reactive (see Somogyi [53] for preparation procedure). The absorbance was measured at 650 nm in a spectrophotometer. The amount of sugars released was determined by comparison with a glucose standard curve with concentrations ranging from 0 to 200 μg mL^−1^.

### Statistics

We used post-hoc analyses (Tukey’s HSD, *P* <0.05) after one-way ANOVA to analyze differences in rhizobial colonization (nodule numbers) and root colonization with AMF in response to different experimental plant treatments (inoculation with live *Colletotrichum gloeosporioides*, *C. gloeosporioides* fragments and treatment with water; controls). Analyses were conducted for two time points: 14 and 21 days after inoculation or fragment application, respectively. T-tests were applied to detect differences in the rate of preliminary colonization with both root symbionts (Figure 1), and the rate of colonization of identically-treated control plants which were part of the subsequent experiments (Figure 5). We also used post-hoc analyses (Tukey’s HSD, *P* <0.05) after one-way ANOVA to analyze differences in enzyme activity (PPO, chitinase, β-1,3-glucanase) after experimental treatments with live *C. gloeosporioides*, *C. gloeosporioides* fragments and water (controls). Lastly, the relationship between enzyme activity and belowground colonization for both microbes was analyzed with Pearson’s two-tailed correlation (Table 1). All statistical analyses were conducted using SPSS (IBM SPSS Statistics 21).

### Availability of supporting data

All the supporting data (raw data on microbial colonization rates and enzymatic activities) are included as additional files.

## Additional files

Additional file 1:
**Plant colonization rates with rhizobia and arbuscular mycorrhizal fungi (AMF).** Raw data for Figure 1. The dataset shows the colonization rates of common bean (*Phaseolus vulgaris*) plants with rhizobia and arbuscular mycorrhizal fungi. Additional file 2:
**Additional independent experiment showing effect of live pathogen and pathogen fragments on root colonization with rhizobia and arbuscular mycorrhizal fungi.** Common bean plants were inoculated with rhizobia and AMF and treated with live *Colletotrichum gloeosporioides* and *C. gloeosporioides* fragments simultaneously. At day 14 and 21 after pathogen treatments colonization of plant roots with **(a)** rhizobia (number of root nodules) and **(b)** percent of roots showing arbuscular mycorrhiza (AMF) colonization were quantified. Values shown are means ± SD; n =9 plants per day. Letters on top of the columns indicate significant differences among pathogen treatment groups according to post-hoc analysis (Tukey’s HSD; *P* <0.05) after one-way ANOVA. Additional file 3:
**Effect of plant treatment with live or fragmented pathogens on defense-associated enzyme activities in leaves.** Raw data for Figures 2, 3 and 4. The data set shows the enzyme activities of foliar polyphenol oxidase, chitinase and β-1,3-glucanase in response to treatment with live or fragmented pathogens (*Colletotrichum gloeosporioides*). (PDF 278 kb) Additional file 4:
**Effect of plant treatment with live or fragmented pathogens on defense-associated enzyme activities in roots.** Raw data for Figures 2, 3 and 4. The data set shows the enzyme activities of polyphenol oxidase, chitinase and β-1,3-glucanase in roots in response to foliar treatment with a live or fragmented fungal pathogen (*Colletotrichum gloeosporioides*). Additional file 5:
**Effect of aboveground treatment with live and fragmented pathogens on belowground microbes.** Raw data for Figure 5 and Additional file 1. The data set shows the colonization rates of *P. vulgaris* roots with rhizobia and arbuscular mycorrhizal fungi (AMF) in plants treated with live a live or fragmented fungal pathogen (*Colletotrichum gloeosporioides*).

## Abbreviations

AMF: Arbuscular mycorrhizal fungi
ASM: Acibenzolar-s-methyl
IG: Iridoid glycosides
IR: Induced response
ISR: Induced systemic response
PPO: Polyphenol oxidase
PR: Pathogenesis-related proteins
SAR: Systemic acquired resistance
