# Supplementary material for: An aboveground pathogen inhibits belowground rhizobia and arbuscular mycorrhizal fungi in Phaseolus vulgaris
Source: BMC Plant Biol. 2014 Nov 28;14:321. doi: 10.1186/s12870-014-0321-4 (PMC4248430; doi:10.1186/s12870-014-0321-4)
Supplement: Additional file 1: — Plant colonization rates with rhizobia and arbuscular mycorrhizal fungi (AMF). Raw data for Figure 1. The dataset shows the colonization rates of common bean (Phaseolus vulgaris) plants with rhizobia and arbuscular mycorrhizal fungi. [file 12870_2014_321_MOESM1_ESM.pdf]

Additional file 1. Plant colonization rates with rhizobia and arbuscular mycorrhizal fungi (AMF)

Day	1	2	3	4	5	6	7	8	9	10	11	12	13	14	15	16	17	18	19	20	21
Nodules (#)																					
	0	0	0	0	0	0	0	0	1	1	0	2	0	3	5	7	15	12	18	22	25
	0	0	0	0	0	0	0	0	2	1	2	2	3	3	4	7	4	13	6	13	10
	0	0	0	0	0	0	0	0	0	0	2	1	2	2	4	8	6	14	13	18	13
	0	0	0	0	0	0	0	0	0	0	2	2	2	3	4	9	8	12	14	11	14
	0	0	0	0	0	0	0	0	0	2	1	1	1	2	3	9	9	11	18	14	18
	0	0	0	0	0	0	0	0	1	1	1	1	2	1	4	12	11	17	15	16	13
	0	0	0	0	0	0	0	0	0	1	0	2	3	5	6	1	12	12	16	14	21
AMF colonization (%)																					
	0	0	0	0	0	0	0	1	1	0	0	1	4	3	6	8	18	19	13	31	34
	0	0	0	0	0	0	0	0	0	1	2	1	3	6	6	8	11	5	15	20	39
	0	0	0	0	0	0	0	1	0	0	2	2	3	2	4	9	14	12	19	18	12
	0	0	0	0	0	0	0	0	2	2	1	2	2	8	8	12	4	9	25	14	11
	0	0	0	0	0	0	0	0	2	2	1	1	1	9	5	3	22	18	26	15	25
	0	0	0	0	0	0	0	0	1	1	1	1	2	3	4	5	10	22	31	27	33
	0	0	0	0	0	0	0	0	0	1	0	2	5	6	10	12	8	14	9	32	41
