# Supplementary figures and images for: An aboveground pathogen inhibits belowground rhizobia and arbuscular mycorrhizal fungi in Phaseolus vulgaris
Source: BMC Plant Biol. 2014 Nov 28;14:321. doi: 10.1186/s12870-014-0321-4 (PMC4248430; doi:10.1186/s12870-014-0321-4)

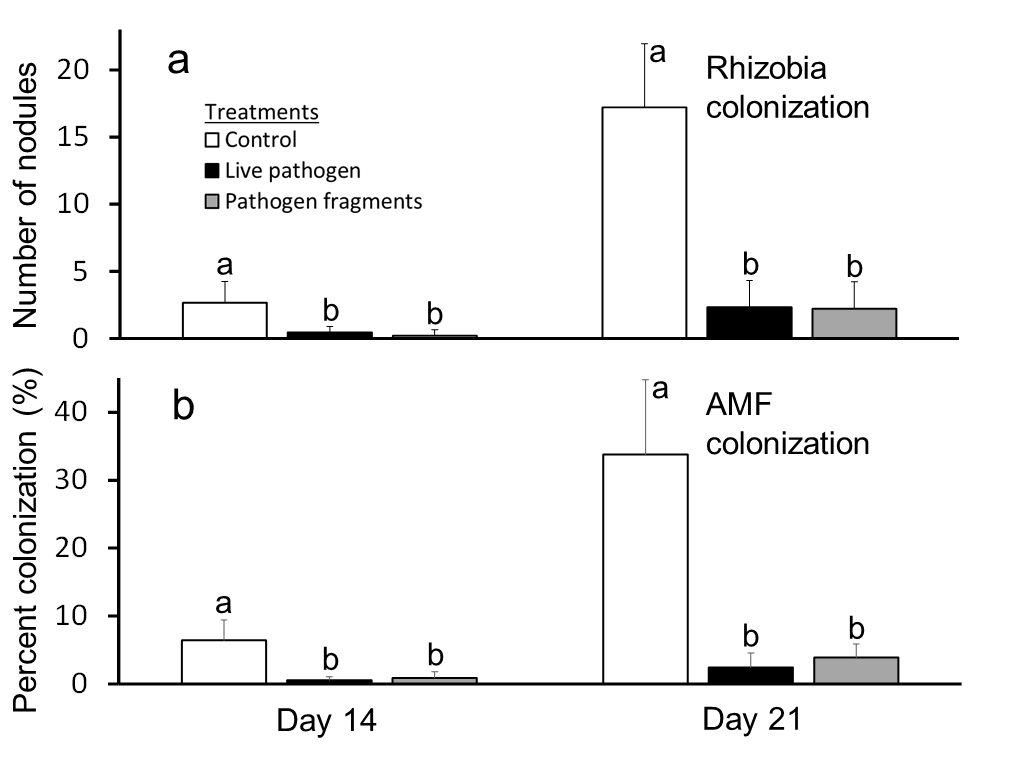

Supplement: Additional file 2: — Additional independent experiment showing effect of live pathogen and pathogen fragments on root colonization with rhizobia and arbuscular mycorrhizal fungi. Common bean plants were inoculated with rhizobia and AMF and treated with live Colletotrichum gloeosporioides and C. gloeosporioides fragments simultaneously. At day 14 and 21 after pathogen treatments colonization of plant roots with (a) rhizobia (number of root nodules) and (b) percent of roots showing arbuscular mycorrhiza (AMF) colonization were quantified. Values shown are means ± SD; n =9 plants per day. Letters on top of the columns indicate significant differences among pathogen treatment groups according to post-hoc analysis (Tukey’s HSD; P <0.05) after one-way ANOVA. [file 12870_2014_321_MOESM2_ESM.tiff]
