# Supplementary material for: An aboveground pathogen inhibits belowground rhizobia and arbuscular mycorrhizal fungi in Phaseolus vulgaris
Source: BMC Plant Biol. 2014 Nov 28;14:321. doi: 10.1186/s12870-014-0321-4 (PMC4248430; doi:10.1186/s12870-014-0321-4)
Supplement: Additional file 3: — Effect of plant treatment with live or fragmented pathogens on defense-associated enzyme activities in leaves. Raw data for Figures 2, 3 and 4. The data set shows the enzyme activities of foliar polyphenol oxidase, chitinase and β-1,3-glucanase in response to treatment with live or fragmented pathogens (Colletotrichum gloeosporioides). (PDF 278 kb) [file 12870_2014_321_MOESM3_ESM.pdf]

Additional file 3. Effect of plant treatment with live or fragmented pathogens on defense-associated enzyme activities in leaves.

Plant #	Treatment (0=control, 1=live pathogen, 2=fragmented pathogen)	Time point after treatment (h)	PPO [μmol O_2 h^{-1} (g fw)$^{-1}$]	Chitinase [Fluorescence units 30 min^{-1} (g fw)$^{-1}$]	β-1,3-Glucanase [μg glucose mL^{-1} (g fw)$^{-1}$]
1	0	0	1.4	30	110
2	0	0	1.3	29	100
3	0	0	1.6	25	96
4	0	0	1.8	31	129
5	0	0	1.5	45	87
6	0	0	1.6	15	95
7	0	0	1.5	25	132
8	0	0	1.6	27	118
9	0	0	1.8	29	98
10	1	0	1.7	33	102
11	1	0	1.1	29	67
12	1	0	1.6	34	137
13	1	0	1.6	39	98
14	1	0	0.9	25	119
15	1	0	1.1	29	88
16	1	0	2.3	16	112
17	1	0	1.6	19	99
18	1	0	1.6	29	115
19	2	0	1.7	18	119
20	2	0	1.8	21	120
21	2	0	2.2	33	87
22	2	0	0.6	32	76
23	2	0	1.6	32	100
24	2	0	0.9	29	97
25	2	0	3.5	25	98
26	2	0	1.1	29	113
27	2	0	0.4	41	132
28	0	24	1.2	29	76
29	0	24	1.3	32	101
30	0	24	1.8	18	97

31	0	24	2.5	22	98
32	0	24	2.5	32	121
33	0	24	1.8	35	118
34	0	24	1.1	27	115
35	0	24	0.6	25	95
36	0	24	0.7	28	118
37	1	24	5.3	25	121
38	1	24	4.2	22	115
39	1	24	1.6	32	134
40	1	24	0.9	26	127
41	1	24	5.1	31	98
42	1	24	4.2	40	87
43	1	24	3.5	21	124
44	1	24	2.7	30	104
45	1	24	3.3	24	111
46	2	24	12.2	45	128
47	2	24	11.2	19	137
48	2	24	13.5	34	125
49	2	24	8.6	25	137
50	2	24	9.4	27	105
51	2	24	12.1	51	97
52	2	24	13.6	53	87
53	2	24	10.0	31	135
54	2	24	10.1	39	120
55	0	48	0.8	25	137
56	0	48	1.1	28	105
57	0	48	1.9	24	97
58	0	48	2.6	32	87
59	0	48	1.1	31	89
60	0	48	1.4	34	99
61	0	48	0.5	26	101
62	0	48	2.2	24	134
63	0	48	1.6	28	112
64	1	48	9.3	31	123

65	1	48	3.3	62	123
66	1	48	7.2	49	145
67	1	48	7.0	41	143
68	1	48	6.2	47	145
69	1	48	2.3	34	112
70	1	48	5.4	71	87
71	1	48	7.2	76	78
72	1	48	8.4	39	123
73	2	48	9.7	51	110
74	2	48	10.5	81	121
75	2	48	10.3	43	129
76	2	48	10.0	39	127
77	2	48	10.2	71	119
78	2	48	9.5	99	99
79	2	48	9.4	65	112
80	2	48	10.4	71	78
81	2	48	10.3	41	120
82	0	72	2.5	22	111
83	0	72	1.7	24	106
84	0	72	0.9	31	104
85	0	72	2.3	33	83
86	0	72	1.5	25	121
87	0	72	1.7	28	106
88	0	72	1.1	34	114
89	0	72	0.7	21	83
90	0	72	0.9	37	115
91	1	72	11.2	49	89
92	1	72	9.2	53	99
93	1	72	11.8	56	123
94	1	72	12.4	58	134
95	1	72	11.1	61	133
96	1	72	8.2	75	127
97	1	72	12.4	69	131
98	1	72	11.5	55	87

99	1	72	13.1	62	86
100	2	72	11.9	110	103
101	2	72	11.2	121	119
102	2	72	8.6	69	105
103	2	72	8.9	42	99
104	2	72	9.2	65	115
105	2	72	9.0	110	121
106	2	72	10.1	66	119
107	2	72	11.1	78	100
108	2	72	7.7	79	104
109	0	96	2.1	26	101
110	0	96	2.2	22	105
111	0	96	1.7	25	99
112	0	96	1.2	25	115
113	0	96	1.2	28	98
114	0	96	1.8	29	119
115	0	96	1.5	31	98
116	0	96	0.8	25	114
117	0	96	1.1	34	121
118	1	96	12.4	72	106
119	1	96	10.3	76	138
120	1	96	13.2	81	92
121	1	96	12.4	59	127
122	1	96	12.1	68	116
123	1	96	10.2	69	125
124	1	96	12.1	77	119
125	1	96	9.6	88	99
126	1	96	13.1	61	78
127	2	96	6.7	79	77
128	2	96	8.2	97	117
129	2	96	6.1	77	117
130	2	96	3.5	98	111
131	2	96	7.9	72	121
132	2	96	6.8	96	106

133	2	96	10.4	72	114
134	2	96	6.2	62	83
135	2	96	9.1	54	125
136	0	120	1.7	29	77
137	0	120	2.9	24	121
138	0	120	2.3	23	106
139	0	120	1.5	27	114
140	0	120	0.9	26	121
141	0	120	1.1	25	87
142	0	120	1.4	31	95
143	0	120	0.5	31	112
144	0	120	1.1	33	118
145	1	120	12.3	81	91
146	1	120	7.2	85	76
147	1	120	7.8	99	132
148	1	120	12.4	61	141
149	1	120	12.1	62	120
150	1	120	8.4	74	111
151	1	120	12.5	105	112
152	1	120	10.7	85	101
153	1	120	12.1	109	111
154	2	120	4.8	57	91
155	2	120	3.4	62	110
156	2	120	8.8	72	96
157	2	120	3.7	101	129
158	2	120	4.7	54	87
159	2	120	5.1	58	118
160	2	120	9.3	69	79
161	2	120	10.5	72	108
162	2	120	6.8	66	121
163	0	144	1.5	25	109
164	0	144	1.1	34	113
165	0	144	2.4	34	67
166	0	144	2.2	32	121

167	0	144	1.6	29	119
168	0	144	1.4	24	109
169	0	144	2.1	26	113
170	0	144	2.5	27	67
171	0	144	0.9	27	121
172	1	144	9.2	82	112
173	1	144	10.3	79	137
174	1	144	8.1	78	101
175	1	144	12.1	104	105
176	1	144	10.4	58	117
177	1	144	9.4	73	126
178	1	144	8.5	71	142
179	1	144	10.5	87	76
180	1	144	9.1	100	87
181	2	144	3.3	29	121
182	2	144	4.7	21	96
183	2	144	10.1	45	121
184	2	144	9.9	34	87
185	2	144	4.1	56	95
186	2	144	4.6	43	112
187	2	144	3.3	46	118
188	2	144	9.2	43	110
189	2	144	4.3	58	102
190	0	168	1.3	32	121
191	0	168	1.4	28	87
192	0	168	1.1	29	95
193	0	168	1.3	31	112
194	0	168	2.1	30	118
195	0	168	0.9	26	95
196	0	168	2.1	24	112
197	0	168	1.7	35	118
198	0	168	1.4	26	120
199	1	168	8.5	54	120
200	1	168	7.2	62	117

201	1	168	8.9	49	109
202	1	168	9.1	73	117
203	1	168	8.5	52	82
204	1	168	9.1	63	97
205	1	168	9.1	68	121
206	1	168	8.3	59	131
207	1	168	6.7	78	91
208	2	168	3.4	34	122
209	2	168	2.7	29	78
210	2	168	3.1	31	109
211	2	168	4.4	45	113
212	2	168	3.5	23	67
213	2	168	3.6	47	121
214	2	168	3.1	31	120
215	2	168	5.1	31	119
216	2	168	4.6	23	99
