# Supplementary material for: An aboveground pathogen inhibits belowground rhizobia and arbuscular mycorrhizal fungi in Phaseolus vulgaris
Source: BMC Plant Biol. 2014 Nov 28;14:321. doi: 10.1186/s12870-014-0321-4 (PMC4248430; doi:10.1186/s12870-014-0321-4)
Supplement: Additional file 4: — Effect of plant treatment with live or fragmented pathogens on defense-associated enzyme activities in roots. Raw data for Figures 2, 3 and 4. The data set shows the enzyme activities of polyphenol oxidase, chitinase and β-1,3-glucanase in roots in response to foliar treatment with a live or fragmented fungal pathogen (Colletotrichum gloeosporioides). [file 12870_2014_321_MOESM4_ESM.pdf]

Additional file 4. Effect of plant treatment with live or fragmented pathogens on defense-associated enzyme activities in roots.

Plant #	Treatment (0=control, 1=live pathogen, 2=fragmented pathogen)	Time point after treatment (h)	PPO [μmol O_2 h^{-1} (g fw)$^{-1}$]	Chitinase [Fluorescence units 30 min^{-1} (g fw)$^{-1}$]	β-1,3-Glucanase [μg glucose mL^{-1} (g fw)$^{-1}$]
1	0	0	1.8	149	194
2	0	0	2.1	125	171
3	0	0	1.6	100	134
4	0	0	2.6	196	189
5	0	0	2.1	114	221
6	0	0	3.1	138	221
7	0	0	1.9	96	189
8	0	0	2.4	147	139
9	0	0	3.2	93	187
10	1	0	1.1	114	134
11	1	0	1.7	95	211
12	1	0	2.1	137	224
13	1	0	2.3	191	154
14	1	0	2.2	137	178
15	1	0	1.8	177	203
16	1	0	2.4	102	189
17	1	0	2.1	103	145
18	1	0	1.7	119	187
19	2	0	2.4	173	200
20	2	0	1.8	172	198
21	2	0	2.7	126	159
22	2	0	2.5	114	176
23	2	0	1.6	88	227
24	2	0	2.7	117	220
25	2	0	2.3	96	149
26	2	0	0.8	137	138
27	2	0	1.1	103	189
28	0	24	2.5	79	199
29	0	24	1.5	131	122
30	0	24	3.5	172	141
31	0	24	2.8	91	157

32	0	24	1.1	154	211
33	0	24	1.9	144	224
34	0	24	3.0	210	184
35	0	24	2.2	124	178
36	0	24	2.4	77	254
37	1	24	1.8	172	200
38	1	24	2.1	156	167
39	1	24	1.1	128	171
40	1	24	2.0	81	134
41	1	24	2.1	96	189
42	1	24	3.2	89	211
43	1	24	2.5	114	154
44	1	24	3.8	121	178
45	1	24	2.1	138	214
46	2	24	3.6	89	201
47	2	24	2.1	98	200
48	2	24	1.3	95	167
49	2	24	1.1	93	178
50	2	24	2.8	121	218
51	2	24	3.7	193	198
52	2	24	2.1	196	120
53	2	24	2.4	138	171
54	2	24	2.2	121	244
55	0	48	3.4	119	213
56	0	48	4.1	79	121
57	0	48	1.2	100	116
58	0	48	4.5	133	125
59	0	48	3.1	91	221
60	0	48	1.3	130	155
61	0	48	2.4	114	187
62	0	48	2.1	172	254
63	0	48	1.9	137	223
64	1	48	3.2	194	111
65	1	48	2.1	144	188
66	1	48	2.9	133	193
67	1	48	2.0	86	200

68	1	48	2.1	84	211
69	1	48	4.2	100	207
70	1	48	7.5	110	244
71	1	48	3.8	107	178
72	1	48	5.4	154	123
73	2	48	3.0	142	201
74	2	48	2.1	119	203
75	2	48	2.1	81	189
76	2	48	1.4	89	145
77	2	48	4.5	130	203
78	2	48	2.5	166	189
79	2	48	4.8	138	145
80	2	48	2.2	128	209
81	2	48	1.7	135	158
82	0	72	1.4	142	204
83	0	72	2.8	135	207
84	0	72	1.5	98	254
85	0	72	2.2	133	154
86	0	72	3.5	103	128
87	0	72	1.9	114	203
88	0	72	2.6	135	189
89	0	72	2.5	142	145
90	0	72	2.9	154	198
91	1	72	8.4	130	168
92	1	72	7.5	146	213
93	1	72	9.6	124	151
94	1	72	7.2	131	175
95	1	72	6.4	84	208
96	1	72	2.9	152	156
97	1	72	5.8	107	224
98	1	72	9.1	103	164
99	1	72	8.0	159	168
100	2	72	2.9	117	159
101	2	72	2.0	117	176
102	2	72	2.1	103	221
103	2	72	2.1	103	220

104	2	72	4.2	144	189
105	2	72	3.2	154	145
106	2	72	2.1	166	163
107	2	72	2.9	142	177
108	2	72	9.6	121	187
109	0	96	2.0	96	155
110	0	96	1.5	114	165
111	0	96	2.1	124	171
112	0	96	1.6	133	232
113	0	96	2.5	102	158
114	0	96	3.1	112	164
115	0	96	2.7	128	173
116	0	96	3.2	149	221
117	0	96	2.1	147	184
118	1	96	10.2	131	211
119	1	96	10.2	110	224
120	1	96	11.3	145	154
121	1	96	11.6	149	178
122	1	96	9.1	100	203
123	1	96	8.5	124	189
124	1	96	8.6	121	145
125	1	96	10.4	102	158
126	1	96	19.6	144	186
127	2	96	8.3	126	156
128	2	96	7.9	121	154
129	2	96	9.9	100	178
130	2	96	8.6	126	213
131	2	96	7.8	144	182
132	2	96	8.2	138	179
133	2	96	9.2	159	191
134	2	96	8.1	133	196
135	2	96	9.1	109	182
136	0	120	2.5	121	214
137	0	120	2.1	114	165
138	0	120	1.8	96	156
139	0	120	1.7	79	212

140	0	120	1.9	131	178
141	0	120	2.5	137	203
142	0	120	2.2	110	143
143	0	120	3.1	142	181
144	0	120	1.7	161	162
145	1	120	9.5	109	162
146	1	120	11.2	128	173
147	1	120	8.8	121	154
148	1	120	8.9	144	178
149	1	120	9.1	116	211
150	1	120	9.1	131	224
151	1	120	9.1	114	154
152	1	120	10.1	107	208
153	1	120	11.3	138	179
154	2	120	11.2	105	213
155	2	120	8.1	95	154
156	2	120	9.5	102	222
157	2	120	11.1	135	154
158	2	120	10.5	152	178
159	2	120	10.2	138	203
160	2	120	9.7	154	188
161	2	120	9.8	158	190
162	2	120	11.4	107	161
163	0	144	2.2	128	173
164	0	144	1.8	98	154
165	0	144	2.4	126	178
166	0	144	2.1	119	203
167	0	144	1.8	168	189
168	0	144	1.4	100	145
169	0	144	2.1	119	168
170	0	144	0.9	123	210
171	0	144	1.5	131	235
172	1	144	6.2	117	178
173	1	144	8.5	114	204
174	1	144	10.2	124	154
175	1	144	7.2	102	178

176	1	144	5.6	119	214
177	1	144	7.2	128	144
178	1	144	7.1	117	213
179	1	144	5.1	130	171
180	1	144	4.8	137	171
181	2	144	9.5	95	154
182	2	144	10.1	100	178
183	2	144	11.4	110	203
184	2	144	6.6	98	154
185	2	144	10.1	133	178
186	2	144	8.6	137	203
187	2	144	8.3	137	189
188	2	144	10.1	133	145
189	2	144	8.1	138	179
190	0	168	2.4	119	148
191	0	168	1.3	114	165
192	0	168	1.1	117	211
193	0	168	2.3	84	214
194	0	168	2.4	124	201
195	0	168	2.2	121	201
196	0	168	2.3	107	154
197	0	168	1.1	149	188
198	0	168	1.4	142	161
199	1	168	4.1	121	154
200	1	168	4.1	107	178
201	1	168	3.1	103	203
202	1	168	4.4	130	189
203	1	168	2.1	116	245
204	1	168	3.1	109	162
205	1	168	3.5	121	169
206	1	168	2.4	124	171
207	1	168	1.8	144	182
208	2	168	8.8	128	173
209	2	168	9.1	126	172
210	2	168	5.8	119	168
211	2	168	7.8	116	165

212	2	168	10.2	114	211
213	2	168	10.1	124	202
214	2	168	8.5	119	211
215	2	168	7.7	137	178
216	2	168	4.2	147	184
