# Supplementary material for: An aboveground pathogen inhibits belowground rhizobia and arbuscular mycorrhizal fungi in Phaseolus vulgaris
Source: BMC Plant Biol. 2014 Nov 28;14:321. doi: 10.1186/s12870-014-0321-4 (PMC4248430; doi:10.1186/s12870-014-0321-4)
Supplement: Additional file 5: — Effect of aboveground treatment with live and fragmented pathogens on belowground microbes. Raw data for Figure 5 and Additional file 1. The data set shows the colonization rates of P. vulgaris roots with rhizobia and arbuscular mycorrhizal fungi (AMF) in plants treated with live a live or fragmented fungal pathogen (Colletotrichum gloeosporioides). [file 12870_2014_321_MOESM5_ESM.pdf]

Additional file 5. Effect of foliar treatment with live or fragmented fungal pathogens (*Colletotrichum gloeosporioides*) on belowground microbes.

Treatment	Plant	Days after treatment	Nodules [#]	AMF colonization [%]
Live pathogen	1	14	0	0
Live pathogen	2	14	0	0
Live pathogen	3	14	0	0
Live pathogen	4	14	0	0
Live pathogen	5	14	0	0
Live pathogen	6	14	2	0
Live pathogen	7	14	0	3
Live pathogen	8	14	0	0
Live pathogen	9	14	0	1
Live pathogen	10	21	2	0
Live pathogen	11	21	6	0
Live pathogen	12	21	3	4
Live pathogen	13	21	0	3
Live pathogen	14	21	0	6
Live pathogen	15	21	4	2
Live pathogen	16	21	3	0
Live pathogen	17	21	1	7
Live pathogen	18	21	0	1
Fragmented pathogen	19	14	0	0
Fragmented pathogen	20	14	1	0
Fragmented pathogen	21	14	1	3
Fragmented pathogen	22	14	1	0
Fragmented pathogen	23	14	0	2
Fragmented pathogen	24	14	0	0
Fragmented pathogen	25	14	0	0
Fragmented pathogen	26	14	0	1
Fragmented pathogen	27	14	0	0
Fragmented pathogen	28	21	5	2
Fragmented pathogen	29	21	1	7
Fragmented pathogen	30	21	3	2
Fragmented pathogen	31	21	0	0
Fragmented pathogen	32	21	0	0
Fragmented pathogen	33	21	6	9
Fragmented pathogen	34	21	4	0
Fragmented pathogen	35	21	2	0
Fragmented pathogen	36	21	0	11
Control (water inoculation)	37	14	4	6
Control (water inoculation)	38	14	2	4
Control (water inoculation)	39	14	2	7
Control (water inoculation)	40	14	1	8
Control (water inoculation)	41	14	3	6
Control (water inoculation)	42	14	4	5
Control (water inoculation)	43	14	3	2
Control (water inoculation)	44	14	4	4
Control (water inoculation)	45	14	4	2
Control (water inoculation)	46	21	12	40

Control (water inoculation)	47	21	19	22
Control (water inoculation)	48	21	25	37
Control (water inoculation)	49	21	13	26
Control (water inoculation)	50	21	9	31
Control (water inoculation)	51	21	16	42
Control (water inoculation)	52	21	17	24
Control (water inoculation)	53	21	22	22
Control (water inoculation)	54	21	12	25

Effect of aboveground treatment with love pathogens and pathogen extract on belowground microbes (timely independent repetition of the above experiment).

Treatment	Plant	Days after treatment	Nodules [#]	AMF colonization [%]
Live pathogen	1	14	0	0
Live pathogen	2	14	1	0
Live pathogen	3	14	0	1
Live pathogen	4	14	0	1
Live pathogen	5	14	0	1
Live pathogen	6	14	1	0
Live pathogen	7	14	0	1
Live pathogen	8	14	2	0
Live pathogen	9	14	0	1
Live pathogen	10	21	3	2
Live pathogen	11	21	1	0
Live pathogen	12	21	2	0
Live pathogen	13	21	0	2
Live pathogen	14	21	3	3
Live pathogen	15	21	2	7
Live pathogen	16	21	7	2
Live pathogen	17	21	2	2
Live pathogen	18	21	1	4
Fragmented pathogen	19	14	0	2
Fragmented pathogen	20	14	1	1
Fragmented pathogen	21	14	0	2
Fragmented pathogen	22	14	0	0
Fragmented pathogen	23	14	0	1
Fragmented pathogen	24	14	0	0
Fragmented pathogen	25	14	0	2
Fragmented pathogen	26	14	1	0
Fragmented pathogen	27	14	0	0
Fragmented pathogen	28	21	2	5
Fragmented pathogen	29	21	3	6
Fragmented pathogen	30	21	1	1
Fragmented pathogen	31	21	1	2
Fragmented pathogen	32	21	1	2
Fragmented pathogen	33	21	1	4
Fragmented pathogen	34	21	3	3
Fragmented pathogen	35	21	7	7
Fragmented pathogen	36	21	1	5
Control (water inoculation)	37	14	6	8
Control (water inoculation)	38	14	1	12

Control (water inoculation)	39	14	2	4
Control (water inoculation)	40	14	2	7
Control (water inoculation)	41	14	4	7
Control (water inoculation)	42	14	3	9
Control (water inoculation)	43	14	2	3
Control (water inoculation)	44	14	3	5
Control (water inoculation)	45	14	1	3
Control (water inoculation)	46	21	24	18
Control (water inoculation)	47	21	21	44
Control (water inoculation)	48	21	18	42
Control (water inoculation)	49	21	11	38
Control (water inoculation)	50	21	13	27
Control (water inoculation)	51	21	18	29
Control (water inoculation)	52	21	14	23
Control (water inoculation)	53	21	23	31
Control (water inoculation)	54	21	13	52
